# Revitalizing agriculture: next-generation genotyping and -omics technologies enabling molecular prediction of resilient traits in the Solanaceae family

**DOI:** 10.3389/fpls.2024.1278760

**Published:** 2024-02-05

**Authors:** Matteo Martina, Valeria De Rosa, Gabriele Magon, Alberto Acquadro, Lorenzo Barchi, Gianni Barcaccia, Emanuele De Paoli, Alessandro Vannozzi, Ezio Portis

**Affiliations:** ^1^ Department of Agricultural, Forest and Food Sciences (DISAFA), Plant Genetics, University of Torino, Grugliasco, Italy; ^2^ Department of Agricultural, Food, Environmental and Animal Sciences (DI4A), University of Udine, Udine, Italy; ^3^ Department of Agronomy, Food, Natural Resources, Animals and Environment (DAFNAE), Laboratory of Plant Genetics and Breeding, University of Padua, Legnaro, Italy

**Keywords:** biotic and abiotic resistance, eggplant, genetic resources, potato, genomic resources, pepper, resilient traits, tomato

## Abstract

This review highlights -omics research in Solanaceae family, with a particular focus on resilient traits. Extensive research has enriched our understanding of Solanaceae genomics and genetics, with historical varietal development mainly focusing on disease resistance and cultivar improvement but shifting the emphasis towards unveiling resilience mechanisms in genebank-preserved germplasm is nowadays crucial. Collecting such information, might help researchers and breeders developing new experimental design, providing an overview of the state of the art of the most advanced approaches for the identification of the genetic elements laying behind resilience. Building this starting point, we aim at providing a useful tool for tackling the global agricultural resilience goals in these crops.

## 1 Introduction

The Solanaceae family comprises significant crops, including, within the most economically and culturally important, tomato (*Solanum lycopersicum*), potato (*Solanum tuberosum*) pepper (*Capsicum annuum)*, and eggplant (*Solanum melongena*), which are cultivated worldwide (FAOSTAT, 2021). Climate changes, such as rising temperatures, altered precipitation patterns, and increased occurrence of extreme weather events, have already demonstrated a significant impact on natural ecosystems and agricultural productivity (Abbass et al., 2022). Therefore, they pose a threat to the distinctive qualities and geographical indications associated with food products.

Over the past few decades, climate change has started impacting Solanaceae crops, and extreme emergent weather patterns are going to significantly affect yield and quality of tomato, pepper, and eggplant (Lee et al., 2018; Bhandari et al., 2021; Suman, 2022; Toppino et al., 2022). While certain agricultural practices and cultivation techniques may provide temporary coping mechanisms, long-term strategies need to be implemented to address the challenges of climate change in vulnerable regions. Breeding strategies play a vital role in developing climate-resilient varieties, and both conventional breeding techniques (CBTs) and new breeding techniques (NBTs) offer powerful tools for enhancing crop resilience within low-input production systems (Razzaq et al., 2021; Xiong et al., 2022). Historically, breeding programs have been focused on developing disease-resistant varieties to ensure sustainable production (Poczai et al., 2022). By selectively breeding for natural resistance or incorporating resistance genes from wild relatives, breeders can enhance the crops’ resilience to common diseases such as late blight, bacterial wilt, and viral infections. Breeding efforts also target agronomic traits that can mitigate the impact of climate change on Solanaceae crops, including drought tolerance, heat tolerance, water-use efficiency (WUE), and nutrient uptake efficiency (NUE). At the same time, enhancing fruit quality attributes is a crucial breeding objective for tomato, pepper, and eggplant (Bebeli and Mazzucato, 2009). For this reason, the main breeding focus has been to improve traits such as flavor, nutritional content, texture, and shelf life, incorporating them into new varieties, ensuring that these crops maintain their appeal to consumers and adapt to changing market demands.

In this paper, the state of the art on next-generation genotyping and -omics technologies for the molecular prediction of multiple resilient traits in the Solanaceae family will be reviewed, with the aim of building a starting point for the research activities within the Recovery and Resilience Facility (RRF) NextGenerationEU founding plan.

## 2 Genetic resources

The Solanaceae Genomics Network (SGN – www.solgenomics.net; Mueller et al., 2005), as well as the G2P-SOL gateway (www.g2p-sol.eu) and Genesys platform (https://www.genesys-pgr.org) are the main centers for genetic and genomic resources for Solanaceae plants, mainly for cultivated species and their crop wild relatives (CWRs). Through these databases, genetic resources can be retrieved, and useful information on their botanical origin and main agronomic characteristics can be collected.

### 2.1 Tomato

Tomato germplasm research has made significant contributions to understanding the genetic and phenotypic diversity at variety level, providing valuable insights for crop improvement of the species through breeding. In one study conducted by Roohanitaziani et al. (2020), a tomato core collection of 122 genebank accessions was extensively explored. The study focused on variation in phenotypic traits, comparing cultivated varieties with wild accessions. The researchers identified key genes and mutations responsible for traits such as fruit size, shape, color, and plant architecture, shedding light on the role of these genes in tomato domestication and breeding, and offering valuable information for precision breeding strategies. Mata-Nicolás et al. (2021) conducted research on a collection of 163 accessions, representing the genetic and morphological variability of tomato from its centers of origin and domestication. This study aimed to explore the genomic landscape of these accessions using sequencing technologies. A substantial number of single nucleotide polymorphisms (SNPs) was observed, and their impact on tomato traits was investigated. The availability of these genomic data, coupled with the creation of F_2_ populations, provides a valuable resource for further genetic studies and the testing of identified SNPs in the context of tomato breeding and selection. In a separate study, Jin et al. (2019) investigated the morphological and molecular diversity of 324 cultivated inbred tomato lines. By analyzing them for 17 agronomic traits, the significant phenotypic variation was assessed, including traits related to disease resistance, fruit characteristics, and plant growth. Genotyping using insertion-deletion (indels) markers further revealed a rich genetic background and the existence of distinct groups within the cultivated tomato lines. This study highlights the potential of employing cluster analysis and molecular markers in guiding parental selection for hybrid breeding and improving tomato cultivars.

### 2.2 Pepper

Pepper germplasm have been extensively investigated by several authors, with each study offering valuable insights into different aspects of genetic diversity and population structure. Lee et al. (2016) focused on exploring the genetic diversity within a large pepper germplasm collection comprising 3,821 accessions. Their investigation revealed that *C. annuum* holds the highest genetic diversity among the 11 species studied. Additionally, they successfully selected a core collection (named CC240), consisting of 240 accessions that represented a wide range of phenotypic variation and displayed higher genetic diversity compared to other core collections. In a study by Zhang et al. (2016), the genetic structure of Chinese pepper germplasm, including both local cultivars and landraces, was examined. By genotyping 372 genebank accessions and analyzing their relationships, they found that the genetic structure corresponded not only to geographic origin, but also to distinct cultivar types. This suggested that migration and human selection played key roles in shaping the genetic diversity of Chinese pepper landraces. Mongkolporn et al. (2015) directed their attention to chili germplasm and aimed to establish a representative core collection. Through the evaluation of 230 chili accessions using microsatellite markers, they highlighted two major groups within the collection. Group I mainly consisted of *C. annuum*, while Group II included various *Capsicum* species, with *C. frutescens*, *C. chinense*, and *C. baccatum* being the most prevalent. This core collection, comprising 28 representative chili accessions, harbored a similar level of diversity to that of the entire collection. Gu et al. (2019) delved into the genetic diversity of pepper accessions in China and investigated the relationship between genetic structure and fruit type, as well as geographic origin. Their findings unveiled the presence of two distinct genetic groups, which aligned with differences in fruit type and geographical distribution, highlighting the impact of both natural and human-driven factors on the genetic structure of pepper germplasm. In a study by Christov et al. (2021), attention was shifted to Balkan pepper accessions. By genotyping 179 C*. annuum* accessions from different locations in the Balkan Peninsula, the authors identified three main clusters based on fruit traits such as shape, size, and pungency. Moreover, a further sub-clustering observed within the main clusters, emphasized a more intricate genetic structure of Balkan pepper germplasm. Nankar et al. (2020) conducted an extensive evaluation of 180 pepper accessions collected from diverse locations across six Balkan countries. Through the examination of agro-morphological traits, fruit quality, and virus resistance, they observed considerable variation and identified six distinct clusters. Their findings indicated the potential of the Balkan pepper collection as a valuable gene source for pre-breeding and cultivar development, particularly those adapted to local conditions. In 2021, Tripodi et al. (2021) conducted a comprehensive genomic analysis of more than 10,000 pepper accessions sourced from genebanks worldwide. Through this investigation, they successfully identified instances of duplicate accessions and taxonomic misassignments, illustrating the intricate challenges associated with classifying interspecific hybrids. Notably, their study unveiled a substantial convergence of pepper types collected across diverse geographical regions, underscoring the global significance of pepper as a cultural commodity. To complement traditional population genetic analyses, the ReMIXTURE method was developed, which effectively quantifies the resemblance between pepper populations originating from different regions. Furthermore, they observed marker-trait associations and selective sweeps affecting pivotal characteristics, such as pungency, which exhibited a nonuniform distribution on a global scale, suggesting a predominant influence of human preferences in shaping the genetic architecture of domesticated pepper varieties. This investigation significantly enriches our understanding of pepper’s genetic history, consequently facilitating advancements in breeding and conservation endeavors. Lastly, Guo et al. (2023) compared the diversity of local landraces and current breeding lines of pepper in China. Through an analysis of qualitative and quantitative traits, traits, as well as molecular markers, they found that current breeding lines exhibited higher diversity in some quantitative traits, especially those related to fruit organs. However, molecular marker analysis revealed that the genetic diversity in breeding lines was lower compared to local landraces. The authors suggested the need for a balanced approach in future breeding processes, focusing not only on target traits but also on molecular marker-based background selection and the incorporation of genetic information from wild and domesticated species.

### 2.3 Eggplant

Research on eggplant germplasm has been conducted by several authors, each shedding light on different aspects of genetic diversity and population structure. Oladosu et al. (2021), in their comprehensive review, emphasized the global occurrences and genebank holdings of cultivated eggplants and their wild relatives. Among such genebanks, the World Vegetable Center (WorldVeg) is the largest public collection of the three cultivated eggplant species (*S. melongena*, *S. aethiopicum* and *S. macrocarpon*), including accessions from related species. As reported by Taher et al. (2017), such material is essential for the development of breeding programs aiming at the enhancement of fruit quality, yield performance, and pest and disease resistance, since wild relatives can harbor undomesticated alleles that can confer novel traits to the commercial varieties. A core collection is a limited set of accessions representing, with a minimum of repetitiveness, the genetic diversity of a crop species and its wild relatives (Frankel, 1984). To this purpose, Naegele et al. (2014) evaluated 99 eggplant genotypes from different landraces, including heirloom cultivars and wild relative species, identifying four different clusters. Successively, by analyzing a dataset of 893 accessions from NARO Genebank, Miyatake et al. (2019) highlighted that the genetic diversity of the Asian accessions, particularly those from South Asian and Southeast Asian countries, was higher compared to other regions, also identifying four distinct clusters (S1–S4), each corresponding to a specific geographical group. Through SPET technology, the collection of 422 accessions, gathered by the G2P-SOL consortium, was genotyped (Barchi et al., 2019a), allowing the identification of duplicates and mislabeled accessions in genebanks. Lastly, Fallahi et al. (2022) assessed the genetic and morphological diversity of eggplant cultivars comprising four Iranian lines and 13 non-Iranian genotypes. They employed morphological analysis of 16 traits and molecular analysis using five SSR markers, revealing significant variations in morphological traits, clear differentiation between eggplants from different origins, and identifying three distinct clusters.

### 2.4 Potato

Cultivated potatoes can be categorized into two primary groups: i) landraces, which are indigenous varieties still cultivated in South America, and ii) improved varieties, grown globally for market production. Potato landraces exhibit significant diversity in tuber shapes, as well as skin and flesh colors. They are cultivated in the upland Andes, spanning from western Venezuela to northern Argentina, and in the lowlands of south-central Chile, with an adaptation to middle to high elevations ranging from 3,000 to 4,000 meters above sea level (Contreras et al., 1993). While genebanks stand as a cost-effective method for conservation, their operations encounter challenges such as potential loss of unique materials and the risk of genetic erosion under suboptimal maintenance or regeneration conditions, and in cases where reorganization is necessitated due to political or environmental factors (Fu, 2017). The management of potato collections varies according to the type of accession, whether it be a variety, breeding line, landrace, or wild species, and the preservation is linked to the corresponding plant organ, including seeds, tubers, *in vitro* plantlets, or shoot tips. The systematic collection of potato genetic resources in South America, encompassing Colombia, Ecuador, Peru, Bolivia, and Chile, was initiated by Russian scientists between 1925 and 1933 (Loskutov and Institute I. P. G. R, 1999). This effort laid the foundation for the first potato germplasm collection at the N. I. Vavilov Institute of Plant Genetic Resources (VIR) in St. Petersburg (Ovchinnikova et al., 2011). Similarly, German scientists undertook collection missions in Chile and Bolivia in 1930, giving rise to the Groß Lüsewitz Potato Collections (GLKS), now integrated into the IPK Federal *ex situ* Gene Bank. This collection comprises 6,300 accessions, including over 2,800 named cultivars, 510 short-day adapted materials, and more than 2,950 accessions of over 130 wild and cultivated *Solanum* species gathered from South and Central America. From 1938 onwards, British expeditions were dispatched to Mexico and South America (Argentina, Bolivia, Peru, Ecuador, Colombia), resulting in the acquisition of 1,164 accessions of wild and cultivated potato species (Hawkes, 1941). This collection formed the basis for the Commonwealth Potato Collection (CPC) currently housed at the James Hutton Institute in Scotland. In the United States, the U.S. Potato Genebank was established in the late 1940s to preclude the importation of varieties that might pose threats to the potato industry or endemic wild species. Since the 1990s, wild potato germplasm has been collected in nearly all Latin American countries (Spooner and Hijmans, 2001). The CGN (Centre for Genetic Resources) potato collection in the Netherlands, originating from Dutch expeditions in 1955 and 1974, was further enriched with germplasm from German missions in 1959, Argentine genebank of INTA-Balcarce, and a collecting mission in Bolivia in 1980 (Van Soest et al., 1983). Collaborative missions between the USA and the Netherlands led to the collection of 2,700 potato accessions from 12 American countries. Presently, approximately 55% of the collection complies with EU plant health requirements for germplasm distribution. Lastly, the International Germplasm Bank for potato was established at the International Potato Center (CIP) in Lima, Peru, during the early 1970s. In collaboration with the Peruvian National Institute for Agricultural Research (INIA), over 300 systematic exploration missions and more than 100 collecting missions were conducted across 12 countries(Huaman et al., 2000; Spooner et al., 2014). In total, 82,293 potato accessions are maintained globally in 89 institutions and four international/regional centers across 59 countries. Among these, 47 institutes in 36 countries hold more than 100 accessions, with just five countries (France, Germany, India, Russia, USA), along with CIP, collectively possessing over 50% of all potato accessions (Nagel et al., 2022).

These findings have significant implications for crop improvement, evolutionary studies, and understanding plant-pathogen interactions. The availability of large collections of genetic resources and advanced genomic technologies has paved the way for further research and the development of improved varieties. The collective efforts of researchers and genebanks worldwide are crucial for the conservation, utilization, and sustainable management of Solanaceae genetic resources to address the challenges of agricultural productivity and food security in the future.

## 3 Genomic resources

Genome sequencing projects have been carried out for several Solanaceae species, serving as fundamental tools for comparative genomics, enabling researchers to identify and analyze genes, regulatory elements, and other functional elements across different species. Moreover, gene expression data and transcriptome analyses have shed light on the dynamic regulation of genes during various developmental stages and in response to environmental stimuli. These resources collectively contribute to a deeper understanding of the genetic diversity, adaptive traits, and agronomically important characteristics of Solanaceae plants, ultimately aiding in the development of improved crop varieties with desirable traits and increased resilience. Moreover, the recent advances in the development of pangenomes allow the overcoming of the limitations imposed using a single genotype as reference, making it possible to discover novel genetic elements.

### 3.1 Tomato

The Tomato Genome Consortium played a pivotal role in presenting a high-quality genome sequence of domesticated tomato (*S. lycopersicum*, cultivar ‘Heinz 1706’; Tomato Genome Consortium, 2012). Later, several upgrades led to the reference version SL4.0 (Hosmani et al., 2019), based on single-molecule sequencing, Hi-C proximity ligation and optical maps. Later, Su et al. (2021) focused on the assembly of an updated reference genome of the tomato cultivar ‘Heinz 1706’ and its comparison with *S. pimpinellifolium* ‘LA2093’. The updated genome assembly (SLT1.0) was 799.09 Mb in length and encompassed 34,384 predicted protein-coding genes, along with 65.66% repetitive sequences. Through comparative analysis, numerous genomic fragments in *S. lycopersicum* that were likely associated with human selection, indicating their potential role in the domestication process of tomato, were identified (Frary et al., 2000; van der Knaap et al., 2014; Pereira et al., 2021). The study conducted by (van Rengs et al., 2022) utilized advanced sequencing technologies, specifically PacBio HiFi and Oxford Nanopore Technology (ONT) long read sequencing, to assemble the genome of an inbred TMV-resistant tomato variety. By combining data from both platforms, highly contiguous and complementary genome assemblies were achieved. The merged assembly, consisting of 12 chromosomes represented as 12 contiguous sequences (N50 = 68.5 Mbp), did not require scaffolding using an orthogonal data type. The merged assembly was validated using chromosome conformation capture data and found it to be consistent with previous tomato genome assemblies that utilized genetic maps and Hi-C technology for scaffolding. This long-read-only assembly provided valuable insights into the structural variations associated with TMV resistance and their impact on the tomato breeding process. While the previously reported study by Roohanitaziani et al. (2020) investigated cultivated tomato accession, Yu et al. (2022) explored the genomes of various tomato species, including *S. habrochaites*, *S. galapagense*, *S. lycopersicum*, *S. pimpinellifolium*, and *S. pennellii*. They focused on genes related to isoprenoid biosynthesis and resistance gene analogs (RGAs). They discovered that the genomes of *S. habrochaites* and *S. galapagense* exhibited an enrichment of genes involved in the terpenoid biosynthetic process. Specifically, they identified a remarkable expansion of the TPS-a subfamily, which encodes sesquiterpene synthases, in *S. habrochaites*, suggesting the potential for diverse or unique sesquiterpene synthesis in this species. Furthermore, they also identified many RGAs in the genomes of all five tomato species, with certain species showing additional RGAs in regions associated with insertion or expansion. These findings highlight the reservoir of resistance genes present in wild tomato relatives acting in networks with varying degrees of complexity, akin to observations in other species and supporting their potential significance in tomato breeding programs. **Table 1** presents a comprehensive list of the reference genomes released for tomato, together with their main characteristics.

**Table 1:** Released reference genomes for the four Solanaceae crops.

Species	Accession	Size (Mb)	N50 (Mb)	Technology	Reference
Tomato	Heinz 1706	782	6,01	PacBio + Hi-C	SL4.0 - Hosmani et al., 2019
		799	17,83	PacBio + Hi-C	SLT1.0 - Su et al., 2021
	Moneyberg-TMV	833	68,5	PacBio HiFi + ONT	MbTMV - van Rengs et al., 2022
Pepper	CM334	3.480	2,5	Short Reads	v1.55 - Kim et al., 2014
	Zunla-1	3.349	1,23	Short Reads	Qin et al., 2014
	UCD-10X-F1	3.212	3,69	10X Linked Reads	Hulse-Kemp et al., 2018
	CA59	2.950	262	Short Reads + PacBio + Hi-C	Liao et al., 2022
	Dempsey	3.053	260,6	PacBio + Optical Mapping + Hi-C	Lee et al., 2022
	Takanotsume	3.058	262,66	HiFi + Optical Mapping + Genetic Mapping	Shirasawa et al., 2023
	Zhangshugang	3.059	259,7	PacBio + Short Reads + Hi-C	Liu et al., 2023
Eggplant	67/3	1.160	92,1	Short Reads + Optical Mapping + Hi-C	Smel v4.0 - Barchi et al., 2021
	HQ-1315	1.170	5,26	Short Reads + ONT + 10X Linked Reads + Hi-C	Wei et al., 2020a
Potato	DM1-3 (2x)	741	17,31	ONT + Hi-C	V6.1 - Pham et al., 2020
	RH89–039-16 (4x)	1.670	1,74	WGS + 10X + ONT + CCS + Genetic Mapping + Hi-C	Zhou et al., 2020
	Otava (4x)	3.100	7,1	PacBio HiFi + HI-C	Hoopes et al., 2022

Assembly size is reported, as well as scaffold N_50_, the applied sequencing technology and the reference.

The pangenome is a recent powerful tool for identifying causal SVs and improving the accuracy of heritability estimates for complex traits (Sherman and Salzberg, 2020; Usadel, 2022). Gao et al. (2019) constructed a pangenome using 725 diverse accessions, identifying 4,873 genes that were not present in the SL3.0 reference genome and revealing PAVs useful for breeding purposes and a rare allele in the *TomLoxC* promoter whose selection apparently modified tomato aroma during domestication. Alonge et al. (2020) described the discovery of thousands of SVs in the tomato genome through the analysis of 100 lines, including 14 new reference-level genome assemblies, showing how SVs can be used to improve tomato crops through genome editing. Furthermore, Zhou et al. (2022) described the construction of a graph pangenome of tomato, by including the genetic information from 838 different tomato lines, and the identification of SVs that are not present in the single linear reference genome. Such pangenome can be used to improve the accuracy of heritability estimates for complex traits, such as fruit flavor and soluble solid content.

### 3.2 Pepper

Pepper reference genome development has been a subject of extensive research, providing valuable insights into the genetic makeup and evolution of this important crop. The first whole-genome sequences of *C. annuum* (‘CM334’) and *C. chinense* (PI159236) were published by Kim et al. (2014). In the same year, Qin et al. (2014) released the genome sequences of the *C. annuum* ‘Zunla-1’ variety and of ‘Chiltepin’ pepper (*C. annuum* var. *glabriusculum*). These studies revealed that the pepper genome is ~3–3.5 Gb in size, contains over 80% repetitive elements, and encodes 35k genes. A resequencing effort (Han et al., 2016) made available the raw sequences of two *C. annuum* lines: ‘Dempsey’, a large bell-type non-pungent genotype, and ‘Perennial’, a pungent genotype with small elongated fruits. In 2017, an improved version of the reference genome of both ‘CM334’ and *C. chinense* ‘PI159236’ was published (Kim et al., 2017), together with the genome sequence of the domesticated *C. baccatum*. This study contributed to deciphering the evolutionary relationships among the three species as well as to estimating the lineage-divergence times occurring in *Capsicum*. In 2018, Hulse-Kemp et al. (2018) obtained the genome sequence of an F_1_ hybrid from a cross between ‘CM334’ with a non-pungent blocky accession of *C. annuum*, by adopting the linked-read sequencing technology. In 2019, Du et al. (2019b) produced low-pass resequencing data from 35 different *C. annuum* lines, to identify a panel of 92 SNPs for population structure analysis. In 2020, Acquadro et al. (2020) conducted whole-genome resequencing of four Italian sweet pepper landraces to gain insights into sequence variation in genes of agronomic value. Some current works (Ou et al., 2018; Lee et al., 2022) developed a pan-genome and a graph-based pangenome approach for pepper, assembling genomes of multiple lines of different *Capsicum* species; these study unveiled informative variants, including presence–absence variants (PAVs), copy-number variants (CNVs), and inversions, and provided insights into the role of transposable elements in shaping the genomic landscape of peppers and insights into *Capsicum* evolution. In 2022, Liao et al. (2022) integrated PacBio long-reads with Hi-C maps with epigenomics, transcriptomic, and genetic variation data to develop a 3D structure of the cultivar “Ca59”. More recently, Shirasawa et al. (2023) presented a study on a Japanese chili pepper landrace (‘Takanotsume’) using long-read sequencing, optical mapping, and genetic mapping techniques to achieve a chromosome-scale genome assembly. Additionally, they conducted a comparative genomics analysis involving two *C. chinense* lines, and 13 genotypes among *Capsicum* species, highlighting nucleotide and structural variations providing valuable information for pan-genomics, breeding, and the understanding of genetic mechanisms underlying important traits in *Capsicum*. **Table 1** contains a list of the reference genomes developed in pepper.

### 3.3 Eggplant

The development of eggplant genomics has been the focus of several research endeavors. In 2014, Hirakawa et al. (2014) presented a study in which they dissected the eggplant genome and constructed a draft genome dataset termed SME_r2.5.1. This dataset covered a significant portion of the eggplant genome, providing valuable insights into its genomic nature. The study estimated that approximately 90% of the gene space was covered, resulting in the prediction of 85,446 genes in the eggplant genome. Comparative analysis of eggplant genes with those of other solanaceous plants and Arabidopsis thaliana revealed the presence of eggplant-specific gene clusters, potentially responsible for conferring unique traits to the crop. Later, Barchi et al. (2019b) made significant contributions to eggplant genomics by obtaining a high-quality genome assembly of eggplant, providing a chromosome-anchored reference. This assembly contained 34,916 genes, confirming the approximate diploid gene number in the Solanaceae family. The study conducted comparative genomic analyses between eggplant and other solanaceous crops such as tomato, potato, and pepper. These analyses highlighted the rapid evolution of miRNA:mRNA regulatory pairs and defense genes within the Solanaceae family. The research also offered insights into the lack of steroidal glycoalkaloid compounds in the *Capsicum* genus. By reconstructing the chromosomal complements of key ancestors, the study shed light on the rearrangements that led to the karyotypes of present-day species and their ancestors. This assembly was enhanced in 2021 by Barchi et al. (2021) through Hi-C retrofitting of the previously released assembly. In 2020, Wei et al. (2020a) released a high-quality reference genome for the eggplant inbred line HQ-1315. This assembly was based on a combination of Illumina, Nanopore, and 10X genomics sequencing technologies, as well as Hi-C technology for genome assembly. The assembled genome exhibited a total size of approximately 1.17 Gb and comprised 12 chromosomes. The study identified various genomic variations, including SNPs, indels, and structural variants (SVs). Furthermore, they developed the first eggplant pangenome, based on 23 cultivated accessions and one accession of *S. insanum* and *S. incanum*, assembling 51.5 additional megabases, and annotating 816 additional genes compared with the reference genome (**Table 1**).

### 3.4 Potato

Sequencing tetraploid plant material, particularly those exhibiting heterozygosity, such as cultivated potatoes, presents a formidable challenge. In addressing this challenge, the haploid potato germplasm DM BARD 1–3 516 R44 (referred to as DM), was employed to construct a reference genome (Veilleux, 2017). The establishment of these monoploids originated from originally heterozygous adapted *S. tuberosum* group Phureja clones, which underwent chromosome doubling procedures (Paz and Veilleux, 1997). After sequencing, the final assembly encompassed approximately 86% of the potato genome, amounting to 844 Mb, and yielded predictions for approximately 39,000 genes. A comparative analysis was then conducted between the sequence of DM and that of the heterozygous diploid potato genome (RH89–039-16, abbreviated as RH), characterized by a pronounced degree of heterozygosity. The alignment between the RH genome and the DM genome revealed a coverage of only 55%, with the occurrence of potentially deleterious mutations being prevalent and identified as a plausible contributor to inbreeding depression (The Potato Genome Consortium, 2011). Trying to further enhance to the genome assembly’s quality and its relevance to potato breeding, a comprehensive marker analysis of a backcross segregation population of DD, a heterozygous clone belonging to the *S. tuberosum* ‘Andigenum Group’, x (DM x DD) was conducted, applying *in silico* anchoring approaches, and integrating data from both physical and genetic maps derived from RH and tomato (Sharma et al., 2013). In 2015, the draft genome of the wild species *S. commersonii*, which diverged from the cultivated potato approximately 2.3 million years ago, was reported. Such assembly, comparable in size to the potato genome at 830 Mb, exhibited markedly reduced heterozygosity, thereby affording valuable insights into evolutionary dynamics and environmental adaptation (Aversano et al., 2015). Further contributions to the understanding of cultivated potato and its wild counterparts were made by Leisner et al. (2018), who sequenced the genome of the diploid inbred clone (M6) of *S. chacoense*. This effort yielded an assembly of 882 Mb and the annotation of 37,740 functional genes. Recent advancements in sequencing technologies have culminated in multiple publications presenting genome assemblies for various diploid and polyploid potato varieties (**Table 1**; Kyriakidou et al., 2020; Pham et al., 2020; Yang et al., 2020; Zhou et al., 2020; Freire et al., 2021; Tiwari et al., 2021; Sun et al., 2022). Finally, two pangenome assembly were obtained through the implementation of long-read sequencing technology: Hoopes et al. (2022) encompassed six potato varieties tailored for distinct markets - fresh (‘Colomba’, ‘Spunta’), chip processing (‘Atlantic’), frozen processing (‘Castle Russet’), and starch markets (‘Altus’, ‘Avenger’), developing the first pan-genome of polyploid potato varieties. In 2023, Bozan et al. (2023) constructed a super-pangenome of 296 diverse potato accessions belonging to the *Solanum* section *Petota*, with a ploidy level ranging from 2x to 5x, constructing a phylogenetic tree using PAVs, and identifying clades for abiotic stress response and flowering and tuberization.

The genome sequencing projects for Solanaceae species, coupled with genetic maps, gene expression data, and transcriptome analyses, have significantly advanced our understanding of their genetic diversity, adaptive traits, and agronomically important characteristics. These resources, along with comprehensive online platforms like the SGN, contribute to the development of improved crop varieties with desirable traits and increased resilience, offering promising solutions for agricultural challenges.

## 4 Genotyping tools and their evolution

The integration of genotyping tools in crop breeding is instrumental in accelerating crop improvement endeavors. By employing tailored techniques and protocols for each crop, breeders can effectively analyze genetic markers, identify desirable traits, and selectively breed plants with superior characteristics. Advances in genotyping make breeding crops faster and more precise, leading to the development of crops with higher yields, better disease resistance, and greater tolerance to environmental stressors. Furthermore, the amalgamation of genotyping data with advanced bioinformatics tools enriches our comprehension of intricate genetic interactions. Consequently, genotyping tools and customized approaches assume a pivotal role in ensuring global food security and addressing pressing agricultural challenges.

### 4.1 Tomato

Genotyping technologies in tomato have advanced significantly over the years, providing valuable insights into the genetic diversity and traits of this important crop. In 2012, Sim et al. (2012) used a high-density SNP array (SolCAP SNP Array) to genotype a collection of 426 tomato accessions, revealing seven sub-populations with distinct genetic characteristics. The study demonstrated the impact of selection on genome variation and identified candidate loci under positive selection. In 2013, Víquez-Zamora et al. (2013) employed next-generation sequencing and high-throughput genotyping to identify a set of 5,528 SNPs, allowing for the evaluation of tomato germplasm at various levels. They discovered markers distinguishing commercial varieties and wild relatives, providing valuable tools for genotyping and phylogenetic analysis. Celik et al. (2017) focused on *S. pimpinellifolium*, a wild species with high breeding potential; using a genotyping by sequencing (GBS) approach, they identified 3,125 unique SNP loci and demonstrated the efficiency of GBS for QTL mapping and introgression of favorable alleles. In 2019, Barchi et al. (2019a) introduced single primer enrichment technology (SPET) for targeted genotyping in tomato and eggplant. Their panel of 5k target SNPs enabled high-throughput genotyping, generating many SNPs, including novel ones, with cross-transferability between related species. Finally, Ziarsolo et al. (2021) presented the K-seq, a cost-effective genotyping methodology based on Klenow amplification and Illumina sequencing. K-seq demonstrated reproducibility and reliability, providing valuable genetic data for diverse species, including tomato.

### 4.2 Pepper

Research on pepper genotyping technologies has made significant strides over the years, contributing to our understanding of genetic diversity and population structure in *Capsicum* spp., also investigating the genetic segregation exploded within several mapping populations (Mimura et al., 2012; Borovsky et al., 2019; Zhu et al., 2019; Lozada et al., 2022; Yi et al., 2022). In 2016, Taranto et al. (2016) employed genotyping by sequencing (GBS) to identify SNPs in a collection of *Capsicum* spp. accessions, with a focus on cultivated pepper (*C. annuum*) genotypes. The GBS analysis resulted in the detection of 108,591 SNP markers, of which 105,184 were specific to *C. annuum* accessions. By further analyzing a subset of 222 C*. annuum* accessions using 32,950 high-quality SNPs, the genetic diversity and population structure of the collection was investigated, and the clustering analysis revealed three distinct clusters, with cluster I encompassing varieties and landraces primarily from Southern and Northern Italy, and Eastern Europe. Clusters II and III comprised accessions from various geographical origins, demonstrating the genetic differentiation of pepper based on both geographical origin and fruit-related features. Moving ahead, Cheng et al. (2016) focused on the development of a high-density interspecific genetic map and the characterization of genetic diversity in pepper. They selected 15,000 SNPs from resequencing data and synthesized an array with 12,720 loci, covering approximately 81.18% of the pepper genome. Using this array, they constructed a high-density genetic map with 5,569 SNPs, utilizing 297 F_2_ individuals. Lastly (Siddique et al. (2022) aimed to understand the genetics of resistance to Pepper yellow leaf curl virus (PepYLCV) in *C. annuum*. By utilizing GBS and constructing a high-density linkage map, they identified three QTLs associated with PepYLCV resistance on chromosomes P1, P7, and P12, respectively. Candidate genes within these QTL regions were inferred, and single markers derived from the QTLs were developed and validated in additional populations and commercial varieties.

### 4.3 Eggplant

Different approaches have been applied also in eggplant, not only investigating natural populations but also analyzing segregation patterns within mapping crosses, as reviewed by Gaccione et al. (2023). In 2012, Barchi et al. (2012) utilized the restriction-site associated DNA strategy combined with high throughput sequencing (RADseq) to develop a large number of functional SNP markers. This approach enabled the construction of a linkage map and the discovery of the genetic basis of anthocyanin-related traits in eggplant. In 2017, Acquadro et al. (2017) used a genotyping by sequencing (GBS) approach to detect SNPs polymorphisms in eggplant, working with a set of 76 accessions of species belonging to the brinjal (*S. melongena*), scarlet (*S. aethiopicum*) and gboma (*S. macrocarpon*) eggplant complexes, to detect the genetic relationships within and between the genepools. Moving to 2019, Liu et al. (2019b) employed the SNP genotyping technique (SNP-seq) to validate genome-wide perfect SNPs in eggplant. This high-throughput SNP genotyping technology allowed a genome-wide association study based on the 219 SNPs identified five associated SNPs located near the SUN and OVATE homologs, which had conserved function in controlling the fruit shape. In the same year, Barchi et al. (2019a) introduced SPET as a targeted genotyping solution, relying. This assay relies on sequencing a region flanking a single primer and simplifies panel design, allowing for higher levels of multiplexing. SPET was used to genotype a panel of target SNPs, both on coding regions and introns/UTRs, in tomato and eggplant, resulting in the discovery of thousands of closely linked, novel SNPs. Lastly, Wei et al. (2020b) utilized specific length amplified fragment (SLAF) sequencing for high-throughput SNP discovery in the eggplant genome. They constructed a high-density genetic map using these SNP markers for QTL analysis of multiple traits in eggplant.

### 4.4 Potato

Analyzing survey data derived from 32 genebanks, it is noteworthy that only two repositories, namely the International Potato Center in Peru and the Raphoe Potato Laboratory in Ireland, have conducted comprehensive genotyping on their respective potato germplasm. In contrast, 20 collections have undergone partial genotyping, and data dissemination has been limited, with only 10 genebanks having made their information publicly accessible thus far (Nagel et al., 2022). Historically, simple sequence repeat (SSR) markers, also identified as microsatellites, have been the predominant choice. together with amplified fragment length polymorphism (AFLP). Nevertheless, a shift toward the adoption of SNPs has been observed, facilitated by genotyping-by-sequencing (GBS) or the deployment of the SolCAP 8K, 12K, or 20K Infinium arrays (Nagel et al., 2022). Distinct regions exhibit unique foci in their genotyping endeavors. For instance, in Asia, specific screening of Japanese germplasm for the potato cyst nematode resistance has taken precedence, with over 1,000 accessions evaluated and potential resistant varieties identified after the initial detection of the nematode in Japan in 2015 (Asano et al., 2021). Conversely, European genebank collections are driven by diverse objectives such as conservation, breeding, and the establishment of reference collections. Consequently, the emphasis in genotyping efforts resides in the assessment of genetic diversity within the established genebanks. A case in point is the Nordic potato collection (NordGen), where 133 potato accessions, varieties, and breeding clones, predominantly cultivated under long daylength conditions in Europe, were genotyped using the Infinium Illumina 20K SNP array; it revealed striking genetic homogeneity, suggesting the need for the introduction of new genetic material for Nordic variety breeding (Selga et al., 2022). Similarly. the Estonian Crop Research Institute has undertaken the fingerprinting of over 450 potato varieties and landraces for the purpose of validating varieties and ascertaining the origin of the material. Employing 8 SSR markers, this endeavor unveiled unique accessions and identified instances of duplicated varieties (Ivanova-Pozdejeva et al., 2022), and the INRAE collection in France sought to develop core collections and facilitate marker-assisted selection, resulting in the fingerprinting of approximately 2,000 accessions using Cleaved Amplified Polymorphic Sequence (CAPS) markers and the SolCAP 8K array. Notably, also the Commonwealth Potato Collection, while only partially genotyped, has been extensively employed in diverse studies encompassing taxonomic and breeding aspects (Hawkes et al., 1994; Bradshaw and Ramsay, 2005; Spooner et al., 2005), as well as evaluations for resistances and tolerances against both biotic and abiotic stress, exemplified by its assessment for resistance to Potato Virus Y (Torrance et al., 2020). The International Potato Center’s germplasm collection has undergone thorough genotyping efforts, with specific findings detailed in (Ellis et al. (2020). Both cultivated and wild potato collections have been subjected to partial genotyping, utilizing techniques such as AFLP and SSR markers. For instance, 1,000 landrace accessions were genotyped (Ghislain et al., 2004), and 742 landraces along with select wild progenitors were also included in this genotyping initiative (Spooner et al., 2007). Moreover, the entire cultivated collection underwent genotyping using the SolCAP 12K array. Intriguingly, a comparative analysis of genotypic information from 250 accessions revealed mismatches between field-maintained plants and *in vitro* clones in slow-growth storage, with a comparable rate of genetic mismatches to other stock centers (Anastasio et al., 2011); a quarter of the cultivated collection was further genotyped using Diversity Arrays Technology sequencing (DarTseq), and approximately 2,000 accessions, spanning both cultivated and wild species, were subjected to genotyping through GBS. Of particular interest is the discernment that the exclusive utilization of data from the SolCAP 12K array can reliably predict the species, aligning with Hawkes taxonomy with a commendable degree of accuracy in North America. A comprehensively reviewed by Nagel et al. (2022), genotyping initiatives have been initiated across diverse collections, albeit with data accessibility remaining a partial facet. In anticipation of future breeding programs, the integration of contemporary technologies such as SolCAP, DArTs, or GBS approaches across entire collections is imperative. Furthermore, the strategic storage of emerging genotypic information in easily searchable databases is crucial for the informed progression of scientific endeavors in the field.

## 5 Classical mapping approaches for biotic and abiotic resistance

Developing mapping populations in horticultural crops holds significant importance for the advancement of plant breeding and genetic research. These populations, created through controlled crosses between selected parental lines, help to identify and locate specific genes or genomic regions associated with traits such as disease resistance, yield, quality attributes, and environmental adaptability. This information not only enhances our understanding of the underlying genetics but also enables breeders to make informed decisions in selecting parental combinations as well as for marker-assisted selection (MAS) for breeding programs to enhance overall crop productivity and sustainability. As resistance traits have been generally eroded by domestication, introgression breeding has emerged as a powerful tool in the field of plant breeding, facilitating the development of crop varieties with improved resistance to abiotic and biotic stresses (Rajpal et al., 2023). Using wild relatives, or related species that have naturally evolved tolerance to both stresses, introgression breeding enables the transfer of favorable traits into crops, broadens the genetic diversity of crop plants and enhances their adaptability and resilience to adverse environmental conditions when interspecific barriers are not present.

### 5.1 Tomato

While hundreds of research articles on the identification of QTLs for quality and productivity traits in tomato have been published in the last decades, starting from Weide et al. (1993), (Eshed and Zamir, 1994), and (Alpert et al., 1995), the focus on biotic and abiotic resistance for QTLs identification has been relatively limited; this could be potentially attributed to the challenges associated with designing robust experimental schemes for evaluating these traits. It is worth noting how, since the 1970s, the genetic diversity of tomato has witnessed a remarkable expansion, primarily due to the implementation of introgression breeding programs involving wild tomato relatives. Notably, the initial surge in genetic diversity occurred through the introgression of resistance traits such tomato mosaic virus (ToMV), southern root-knot nematode (*Meloidogyne incognita*), and leaf mold disease (*Cladosporium fulvum*) from *S. pennelli* and *S. pimpinellifolium*. These introgressions ranged in size, with some spanning approximately 5% of a chromosome (such as the introgressions of Cf-4 and Cf-9 on chromosome 1, conferring resistance to *C. fulvum*), while others covered half of chromosome 9 (ToMV). Collectively, these introgressions significantly enhanced the overall genetic diversity. A subsequent wave of diversity, starting in late ‘80s, had a dual impact on fruit size and quality traits (Schouten et al., 2019). One of the earliest studies identifying resistance QTLs to biotic stress (Bai et al., 2003) investigated the resistance of tomato to powdery mildew caused by *Oidium lycopersici*, reporting three QTLs on chromosomes 6 and 12. Panthee et al. (2017) focused on late blight resistance caused by *Phytophthora infestans*; in this work, the major genes (Ph-1, Ph-2, and Ph-3), along with additional minor QTLs associated with resistance, were successfully mapped in an inter-specific cross (*S. lycorpersicum* x *S. pimpinellifolium*). Qi et al. (2022) focused on Tomato spotted wilt virus (TSWV) resistance and fine-mapped the NLR gene Sl5R-1, which regulates resistance to TSWV, by identifying a gene cluster consisting of three genes (Sl5R-1, Sl5R-2, and Sl5R-3) that code for NBS-LRR proteins, along with the transcription factors regulating the resistance mechanism. Moving to abiotic stress resistance, Arms et al. (2015) explored the tolerance of wild tomato (*S. habrochaites*) root chilling, identifying a major QTL (stm9), on chromosome 9, which controlled shoot turgor maintenance under root chilling. Successively, Wen et al. (2019) identified five major QTLs related to heat tolerance and identified candidate genes within these QTLs using RNA-seq analysis. **Table 2** shows a list of loci that have been associated with candidate genes for resistance to abiotic and biotic stress in tomato.

**Table 2:** List of QTLs and potential candidate genes for resistance to abiotic and biotic stress in the four Solanaceae.

Species	Stress	Stressor	QTL	Chr.	Position (Mb)	Potential Candidate Genes	Genome	Reference
Tomato	Biotic	TY-1	*Ty-1*	6	34.35-34.37	*Ty-1*	SL2.5	Zamir et al., 1994
		Bacterial Canker	*Rcm6*	6	37.24-41.15	*RLK/RPL and NLR*	SL3.0	Abebe et al., 2022
		Colorado potato beetles	*-*	6	45.62-47.49	*GAME9*	SL2.5	Vargas-Ortiz et al., 2018
		Leaf Mold	*Cf-10*	1	3.35-3.74	*TMK1*	SL3.0	Liu et al., 2019a
		Late Blight	*Ph-3*	9	71.29-71.94	*Ph-3*	SL2.5	Panthee et al., 2017
		Bacterial spot	*RX4*	11	53.54-53.57	*Rx4*	SL2.5	Pei et al., 2012
	Abiotic	High Temperature	*qCC-1-5*	1	81.64-86.37	*SlGST, SlUBC5 and SlARG1*	SL2.5	Wen et al., 2019
			*qHII-2-1*	2	38.98-40.85	*SlCathB2*		
		Root Chilling	*stm9*	9	1.82-2.05	*Solyc09g8440 -…90*	SL2.5	Arms et al., 2015
Pepper	Biotic	Bacterial Wilt	pBWR-1	1	47.7-161.1	*PR-1 and RPM1s*	CM334	Chae et al., 2022
		Bacterial Wilt	qRRS-10.1	10	192.3-195.3	*Bs2s, PR-1, LURPs*	CM334	Du et al., 2019a
		Potyvirus Mosaic Diseases	*Pvr4*	10	230-233	*Pvr4 and R-genes*	CM334	Devran et al., 2015
Eggplant	Biotic	Fusarium wilt	FomCH11	11	63-71	*RPP13s and R1C-3s*	‘67/3’ (v3)	Tassone et al., 2022
			FomCH02	S67320	–	*RES1*	asm_305	
		Bacterial wilt	EBWR9	9	69.4-71.17	*NBS-LRR and RLK genes*	‘67/3’ (v2.5)	Salgon et al., 2017
Potato	Biotic	Late Blight	MQTL_1_Late_blight	4	–	*R2-like gene cluster*	–	Danan et al., 2011
			MQTL_2_Late_blight		–	*RB cluster*	–	
			*dPI09c*	9	60.65-61.04	*R8*	DM	Jiang et al., 2018
		Cyst nematode	*Grp1*	5	–	NBS-LRR gene cluster	–	Finkers-Tomczak et al., 2009
	Abiotic	Drought	DRYM	3	47.42-57.31	BAK1, RGL1, GID1…	DM	Schumacher et al., 2021

### 5.2 Pepper

As explained, wild relatives are the best reservoir of resistance alleles from which a breeder can draw, and have been used since mid ‘90s to introgress resistances in the cultivated pepper genetic background (Lefebvre et al., 1995). However, interspecific crosses could be hard to obtain, according to species, and in pepper hybridization is usually complicated by interspecific barriers, which can preclude crossing within the *Capsicum* genus (Parry et al., 2021). Thus, searching for alleles already available in the *C. annuum* genetic background has represented a valuable option since early 2000 (Ben-Chaim and Paran, 2000). Intraspecific mapping populations have been applied also for resistance breeding: Eun et al. (2016) searched for cucumber mosaic virus (CMV) resistance in *C. annuum*, identifying two QTLs accounting for a notable proportion of phenotypic variation within the mapping population, and providing valuable information for the development of CMV-resistant pepper cultivars. More recently, Du et al. (2019a) aimed to uncover the genetic basis of pepper’s resistance to bacterial wilt. Their findings demonstrated that resistant rootstocks effectively suppressed the spread of *Ralstonia solanacearum* into the scion. Through specific-locus amplified fragment sequencing and bulked segregant analysis, two adjacent resistance-associated regions on chromosome 10 were identified. Anthracnose, a destructive disease affecting pepper crops, was the focus of investigation by Zhao et al. (2020). The study aimed to identify the gene responsible for anthracnose resistance in pepper through re-sequencing genotyping. The anti-anthracnose locus AnRGO5 was mapped to a specific region on the P5 chromosome, in a region containing five genes. These findings provide valuable information for future studies on the genetic basis of anthracnose resistance and marker-assisted selection in pepper breeding. Sun et al. (2020) focused on aphid resistance in *Capsicum baccatum*. The study conducted a QTL analysis for *Myzus persicae* resistance and identified two QTLs on chromosome 2, affecting aphid survival and reproduction. Fine mapping narrowed down the major QTL*, Rmprp-1*, to a genomic region encoding analogues of resistance genes belonging to the leucine-rich repeat domain (LRR-RLKs) family. The research conducted by van Haperen et al. (2021) focused on thrips resistance in *Capsicum*, fine-mapping a major QTL for thrips resistance on chromosome 6 and identifying 15 candidate genes within the QTL region. Lastly, in Chae et al. (2022), the genetic regions associated with bacterial wilt (BW) resistance in pepper crops were explored. Through QTL analysis, genomic loci and alleles critical for BW resistance were identified. Genotyping-by-sequencing was employed, resulting in the construction of a pepper genetic linkage map revealing the presence of a significant QTL, pBWR-1, on chromosome 1, explaining a substantial proportion of phenotypic variance. As the main findings on biotic resistance in pepper have been extensively reviewed by Parisi et al., 2020, including several lists of alleles donors and identified QTLs, **Table 2** presents a brief overview of the latest findings in pepper genetic resistances.

### 5.3 Eggplant

Recently, Toppino et al. (2022) comprehensively reviewed the state of the art of the eggplant research on introgression breeding for resistances trait, reporting that, while strides have been made in mapping traits related to fruit features and biotic stress resistance, the full potential of marker-assisted breeding for a wider range of abiotic and biotic stresses remains largely unexplored. The availability of introgression lines carrying stress-resistance genes from wild relatives like *S. linneanum* (Doganlar et al., 2002; Frary et al., 2003) *and S. incanum* presents a promising avenue for uncovering chromosomal regions responsible for conferring tolerance, but further research and population screening hold the potential to unveil these mechanisms and contribute to the development of more robust and resilient eggplant varieties. **Table 2** reports the identified QTLs for biotic and abiotic resistance for which candidate genes have been identified.

### 5.4 Potato

Several wild potato species has been reported to be suitable as introgression material, and have been used for mapping since early ‘90s (Barone et al., 1990; Kreike et al., 1993), both for biotic and abiotic resistances. Among them, seven species have been classified as primary gene pool (i.e. closely related species that can be generally crossed to cultivated varieties), 62 as secondary gene pool, and 24 as tertiary gene pool (Castañeda-Álvarez et al., 2015). Moreover, the species belonging to the primary and secondary gene pool are the mostly used for breeding and introgression of favorable traits (Maxted et al., 2012). The species belonging to the primary gene pool have been reported to be suitable for introgressing a number of desirable alleles, including frost tolerance (*S. acaule*), blackleg and soft rot resistance (*S. berthaultii* and *S. candolleanum*), *Verticillium* wilt (*S. berthaultii and S. vernei*), *Globodera pallida*, and cyst nematode (*S. brevicaule* and *S. vernei*) resistance, while the ones belonging to the secondary pool have been used as introgression material for late blight resistance (*S. stoloniferum*, *S. raphanifolium*, *S. demissum* and *S. verrucosum*), Potato Virus Y resistance (*S. palustre*), Potato Leaf Roll virus resistance (*S. demissum*), drought and heat tolerance (*S. chacoense*), frost tolerance (*S. boliviense*), blackleg and soft rot resistance (and *S. hjertingii)*, and; *Verticillium* wilt resistance (*S. chacoense*; Nagel et al., 2022). For example, Dong et al. (2023) delved into the abiotic challenge of frost tolerance in potatoes. By constructing a BC_1_ population from cold-resistant (*S. commersonii*) and cold-sensitive (*S. verrucosum*) wild species, the study identified four QTLs on chromosomes 2 and 11 associated with frost tolerance. The inclusion of bulked segregant analysis and identification of candidate genes adds depth to the understanding of the complex genetic architecture governing frost resistance in potatoes under varying low-temperature conditions. Not only wild species, but also commercial material can be a reserve for resistance and tolerance alleles. Santa et al. (2018)focused on addressing the phytosanitary challenges posed by late blight and Guatemalan potato tuber moth in Colombia and Ecuador. By utilizing a F_1_ tetraploid segregant population, they successfully identified six QTLs linked to resistance against *Phytophthora infestans* and four QTLs related to *Tecia solanivora*. The genetic linkage map constructed demonstrated good distribution across the genome, providing valuable insights for breeding programs in regions where these pests limit potato production. Odilbekov et al. (2020) concentrated on early blight caused by *Alternaria solani*, causing an economic significant foliar disease in potato-growing regions. Through the characterization of a bi-parental tetraploid population, the study identified several QTLs associated with resistance to early blight in both leaves and tubers. Notably, the absence of a statistically significant correlation between resistance scores in leaves and tubers highlights the complexity of the genetic basis for this trait. In 2021, da Silva Pereira et al. (2021) addressed the common scab caused by *Streptomyces* spp., an understudied bacterial disease affecting the potato industry, identifying a QTL on chromosome 3 contributing to disease resistance, providing valuable information for genomics-assisted breeding approaches. Collectively, these studies contribute significantly to the development of resistant cultivars, addressing both biotic and abiotic challenges faced by potato crops globally.

## 6 Modern approaches for the identification and selection of resilience alleles

In recent years, several innovative approaches have been developed for the identification and selection of resilience alleles (Magon et al., 2023; Villalobos-López et al., 2022), enabling a deeper understanding of the genetic basis of resilience in various organisms. Transcriptomics have played a crucial role. By comparing the transcriptomes of resilient and susceptible individuals, candidate genes involved in such traits can be identified. Additionally, modern mapping approaches such as genome wide association (GWA) studies and bulked segregant analysis (BSA) combined with sequencing technologies have proven effective in identifying genetic markers associated with resilience traits. These approaches provide insights into the specific genomic regions and alleles contributing to plant adaptability. Furthermore, Genomic Prediction leverages genomic data to predict the resilience potential of individuals, facilitating targeted breeding programs. Finally, the integration of phenomics, which involves high-throughput phenotypic data collection, enables the comprehensive characterization of resilience traits and their associations with genomic information. The combination of all these approaches offers promising avenues for accelerating the identification, selection, and breeding of resilient individuals across various organisms, ultimately contributing to the development of more resilient and sustainable agricultural and ecological systems.

### 6.1 Transcriptomics for abiotic/biotic resilience-related genes identification

Transcriptomics is a powerful tool for identifying candidate genes associated with abiotic and biotic resilience. By analyzing gene expression patterns, researchers can uncover genes involved in tolerance to drought, heat, salinity, pathogen resistance, and pest resistance. Comparative transcriptomic analyses between resilient and susceptible individuals enable the identification of differentially expressed genes as potential candidates, without the need for developing and testing complex mapping populations. These findings have significant implications for enhancing crops’ resilience, but further research is usually needed to characterize the precise roles of these candidate genes and develop targeted strategies for resilience enhancement. As set of examples, here we collected a sample of the latest research applying RNAseq for the identification of abiotic and biotic resilience-related genes in the four crop Solanaceae.

#### 6.1.1 Tomato

- Ashrafi-Dehkordi et al. (2018) conducted a meta-analysis of gene expression integrating data from 391 microarray samples derived from 23 different experiments, identifying 2,336 differentially expressed genes (DEGs) involved in multiple stresses. Overall, 361 DEGs exhibiting conserved expression patterns between biotic and abiotic stresses, indicated some level of shared mechanisms in response to different stressors, but, in general, the number of genes differentially regulated in response to biotic stresses (1,862) far exceeded those regulated by abiotic stresses (835) gene were categorized and some enrichments on transcription factor (TF) families were spotted, emphasizing the crucial role of TFs in orchestrating stress-responsive gene expression. In a more recent study, Zhou et al. (2019) focused specifically on the combined effects of cold and drought stress in tomatoes, investigating the physiological and genetic tomato responses. The study found that the combined stress condition altered physiological parameters such as water loss rate, H2O2 content, and hormone levels (e.g., ABA, IAA, ZR, melatonin), highlighting the close interaction between physiological and genetic responses, and the related complex regulatory mechanisms. More recently, Amoroso et al. (2023), resembling the work by Ashrafi-Dehkordi et al. (2018) but going further the limits of microarray technologies, applied RNA-seq for the investigation of tomato plants exposed to seven biotic stresses; the authors included *Cladosporium fulvum*, *Phytophthora infestans*, *Pseudomonas syringae*, *Ralstonia solanacearum*, *Sclerotinia sclerotiorum*, Tomato spotted wilt virus (TSWV), *Tuta absoluta*, and five abiotic stresses (namely drought, salinity, low temperatures, and oxidative stress). Following the analysis, they discovered a total of 1,474 DEGs that were commonly shared between biotic and abiotic stresses. Among these, 67 DEGs exhibited response patterns in at least four different stress conditions, signifying their pivotal roles in stress adaptation. The identified DEGs encompassed a diverse set of genes associated with stress response pathways. Notable examples included Receptor-Like Kinases (RLKs), Mitogen-Activated Protein Kinases (MAPKs), Fasciclin-like arabinogalactans (FLAs), glycosyltransferases, genes involved in auxin, ethylene (ET), and jasmonic acid (JA) pathways, as well as MYBs, basic leucine zippers (bZIPs), WRKYs, and Ethylene Responsive Factor (ERF) genes. All the latter gene families play crucial roles in mediating signal transduction, hormone regulation, and transcriptional reprogramming during stress response. The identified DEGs hold immense potential for further exploration using biotechnological approaches to enhance plant tolerance in real-world agricultural settings.

#### 6.1.2 Pepper

Li et al. (2016) explored through RNAseq the role of brassinosteroids (BRs) and in particular of 24-epibrassinolide (EBR), during chilling stress. By supplementing EBR under low temperatures, the study identified some DEGs, likely regulated by EBR, associated with photosynthesis, hormone metabolism, redox homeostasis, signaling pathways, transcription, and defense. The results highlighted the synergistic crosstalk between BRs and other signaling pathways, such as salicylic acid (SA), jasmonic acid (JA), and ethylene (ETH), occurring after chilling. More recently, Zhang et al. (2022b) focused on deciphering the molecular mechanisms underlying pepper’s response to cold stress. They conducted transcriptomic and metabolomic analyses on two pepper cultivars, XS (cold-sensitive) and GZ (cold-resistant), identifying key players for the response to cold stress. López-Serrano et al. (2021) compared the transcriptomic responses of a salt-tolerant accession (A25) and a salt-sensitive accession (A6) to NaCl stress. They identified DEGs and pathways associated with salt stress tolerance, highlighting how the up-regulated in the tolerant genotypes were involved in osmotic potential regulation, ion homeostasis, growth regulation, photosynthetic protection, antioxidant activity, and stress signaling. Notably, the ABA signaling pathway played a prominent role in salt tolerance, with key genes involved in ABA biosynthesis and signaling being upregulated in the salt-tolerant accession. Ma et al. (2023) investigated the alleviation of salt stress in pepper through the application of SA through aa transcriptomic analysis The study revealed that SA treatment regulated the expression of genes involved ion absorption and homeostasis, redox regulation, stress signaling, and transcriptional control. The authors suggested that SA acts as a key modulator in enhancing pepper’s tolerance to salt stress by orchestrating multiple physiological and molecular responses. Borràs et al. (2021) examined drought stress effects on bell pepper, identifying two NAC transcription factors (CaNAC072 and CaNAC104) with contrasting responses. A validation approach through gene silencing was attempted, confirming the role of CaNAC072 in enhancing drought tolerance, while not for CaNAC104. Lastly, Padilla et al. (2023) focused on understanding the tolerance mechanisms of NIBER^®^, a water stress and salt-tolerant pepper hybrid rootstock compared to a sensitive pepper accession (A10) to short-term water stress. Under water stress, NIBER^®^ exhibited up-regulation of transcription factors like DREBs and MYC, as well as increased levels of auxins, abscisic acid (ABA), and jasmonic acid (JA). The rootstock also showed an increase in osmoprotectant sugars and antioxidants, along with enhanced expression of aquaporins and chaperones. These findings indicate the strategies employed by NIBER^®^ to overcome water stress, involving osmotic regulation, hormone signaling, and stress response pathways, and offer potential targets for enhancing water stress tolerance in pepper.

#### 6.1.3 Eggplant

Recently, Gaccione et al. (2023) comprehensively reviewed the state of the art of transcriptomic research for biotic and abiotic stress in eggplant, combining such information with QTLs data, when possible, with the aim of identifying potential candidates for eggplant resilience. For instance, QTLs and candidate genes associated with bacterial wilt resistance were organized in quantitative genomic regions (QGRs) associated with resistance traits on chromosome 4 and 1. For resistance to R. solanacearum, SmMYB44 (SMEL4.1_04g019540.1), on chromosome4, was reported to lead to spermidine accumulation and resistance genotypes. For fungal wilt resistance, QGRs were identified on chromosome 2 and 11, were Dirigent protein 23 have been associated with the FomE02.01 resistance locus.

#### 6.1.4 Potato

Chandrasekar et al. (2022) investigated the impact of potato cyst nematodes (PCNs), a significant pest, on the resistant cultivar ‘Kufri Swarna’ and the susceptible cultivar ‘Kufri Jyoti’. Employing high-throughput RNA sequencing, the study revealed 791 differentially expressed genes (DEGs) in the resistant cultivar, including upregulated disease resistance genes (KIN) and transcription factors (WRKY, HMG, and MYB), as well as enriched pathways like mitogen-activated protein kinase (MAPK) signaling. In 2023, Agho et al. (2023) assessed the field resistance of the Estonian potato cultivar ‘Ando’ to *Phytophthora infestans*, compared with the cultivar ‘Arielle’, identifying 5927 DEGs in the first on variety, and 1161 DEGs in the second one. Compared with ‘Arielle’, signal transduction, endocytosis, MAPK, autophagy, serine/threonine kinase activity, and plant-pathogen interaction pathways were reported to be significantly upregulated in ‘Ando’. Similarly, investigating the transcriptomic response to abiotic stress, Li et al. (2020) focused on the response of the salt-tolerant genotype ‘Longshu No. 5’ to salt stress. The study identified 5,508 DEGs and highlighted the significant enrichment of categories related to nucleic acid binding, transporter activity, ion/molecule transport, ion binding, kinase activity, and oxidative phosphorylation. More recently, Jian et al. (2022) and Tang et al. (2023) delved into the molecular responses of potatoes to drought stress and ubiquitination, respectively. Jian et al. identified 12,798 DEGs in response to simulated drought stress, elucidating pathways such as galactose metabolism, fatty acid metabolism, and plant-pathogen interaction. Tang et al. comprehensively analyzed deubiquitinase (DUB) genes under PEG-induced drought stress, revealing 6,067 down-regulated and 4,950 up-regulated DEGs, with DUBs implicated in the regulation of osmotic stress.

Collectively, these studies have significantly advanced our understanding of the molecular mechanisms underlying Solanaceae’s resistance to biotic and abiotic stresses. By employing transcriptomic approaches, researchers have identified differentially expressed genes, enriched gene ontologies, and key molecular pathways associated with stress responses. Continued research in this field will definitively contribute to the development of increasingly sustainable agriculture.

### 6.2 Association studies

Effectively harnessing the potential of crop germplasm collections necessitates a full comprehension of the genetic diversity contained within them. Such understanding allows the identification of essential “core collections,” which consist of a limited number of accessions that represent as much diversity as possible. These core collections hold immense significance in evolutionary biology and breeding, playing a crucial role in planning phenotypic characterization, seeking genetic resources adapted to varying environmental conditions, selecting materials for breeding programs and hybridization experiments, as well as guiding the selection of accessions for genome sequencing and comparative studies. The advent of NGS technologies allowed the exploitation of GWAS (genome-wide association study) as a routine practice in uncovering associations between genotype and phenotype across many species. GWAS serves as an alternative to traditional bi-parental linkage mapping, enabling the dissection of quantitative traits in crops. Within the framework of GWAS, the prevalence of polymorphisms is influenced by the overall genetic variation present within the germplasm panel. Similarly, the non-random associations between genetic markers, known as linkage disequilibrium, are influenced by factors beyond recombination frequency, including genetic drift, the plant’s mating system, and historical selection events. It is worth noting that population structure can introduce misleading connections between potential markers and phenotypes, especially when there are variations in trait means or frequencies across subpopulations. However, understanding the population structure within germplasm collections can help mitigate this risk.

#### 6.2.1 Tomato

Since the first GWAS in tomato (Sauvage et al., 2014),several investigations have been conducted to investigate various aspects of tomato genetics and resistance to diseases. For example, Nguyen et al. (2021) investigated the genetic factors associated with resistance to bacterial wilt (BW) in tomato. Their research focused on a core collection of 191 tomato varieties and employed the 51 K Axiom^®^ tomato array for genotyping. The team identified eight marker-trait associations (MTAs) linked to BW resistance, with two consistently observed on chromosomes 4 and 12. These QTLs explained a significant proportion of the observed variations. Notably, the study successfully demonstrated the effectiveness of the identified SNP markers in distinguishing resistant tomatoes within commercial cultivars. Additionally, the researchers identified four MTAs specific to disease assays, implying the existence of environment-specific QTLs on chromosomes 1 and 8–10. More recently, Park et al. (2022) identified novel QTLs for Powdery Mildew Resistance (PMR) through a GWA approach. They discovered five novel QTL regions associated with PMR, clearly distinct from previously reported ones, and identified three promising candidate genes (two nucleotide binding site-leucine rich repeat class genes and a receptor-like kinase gene).

#### 6.2.2 Pepper

GWA studies have been performed also in pepper, identifying mainly agronomical and quality traits like fruit shape, fruit size, and male sterility (Nimmakayala et al., 2016; Colonna et al., 2019; Wu et al., 2019; Wu et al., 2022). While Potnis et al. (2019) identified loci associated with defoliation in response to infection with *Xanthomonas gardneri* in the USDA core collection, (Ro et al., 2022a) focused on Phytophthora blight resistance in pepper and performed a GWAS to identify SNPs associated with resistance to *Phytophthora capsici* and identifying by high-resolution melting (HRM) two markers: Chr02-1126 marker predicted *P. capsici* resistance with 78.5% accuracy, while the QTL5-1 marker predicted resistance with 80.2% accuracy.

#### 6.2.3 Eggplant

In the same way, few GWAS analyses have been conducted in eggplant, mainly on agronomical and quality traits (Cericola et al., 2014; Portis et al., 2015; Arrones et al., 2022; Ro et al., 2022b), but studies focusing on resistance traits have not been published yet. On the other hand,

The results from these studies, especially when sustained by QTL analysis data, provide useful information for the understanding of the genomic regions mainly involved in resistance traits, and might be targeted in future experiments to depict candidate genes and understand the genetic mechanisms regulating tolerance in high-value genotypes within core-collections.

#### 6.2.4 Potato

As for other species, several studies have identified SNPs associated with agronomical and quality traits, such as tuber shape, eye depth, and fry color retention during low-temperature storage (Sharma et al., 2018; Byrne et al., 2020; Zhang et al., 2022a; Pandey et al., 2023; Zhao et al., 2023). Moreover, multiple genetic markers and candidate genes aimed at enhancing disease resistance to late blight have been identified by Wang et al. (2021) and Sood et al. (2023), while Díaz et al. (2021) and Alvarez-Morezuelas et al. (2023) investigated plant’s response to abiotic stressors, revealing genes and QTLs that may aid in developing varieties better suited to changing environmental conditions and drought seasons. Additionally, Vos et al. (2022) identified two haplotypes (namely SGT1 and SGT2) as strictly associated with the α-solanine and α-chaconine, two steroidal glycoalkaloids— plant defense molecules with anti-nutritional properties for humans— that interact play a key role in plant’s defense against pathogens.

### 6.3 Innovative mapping approaches

**Figure 1:**
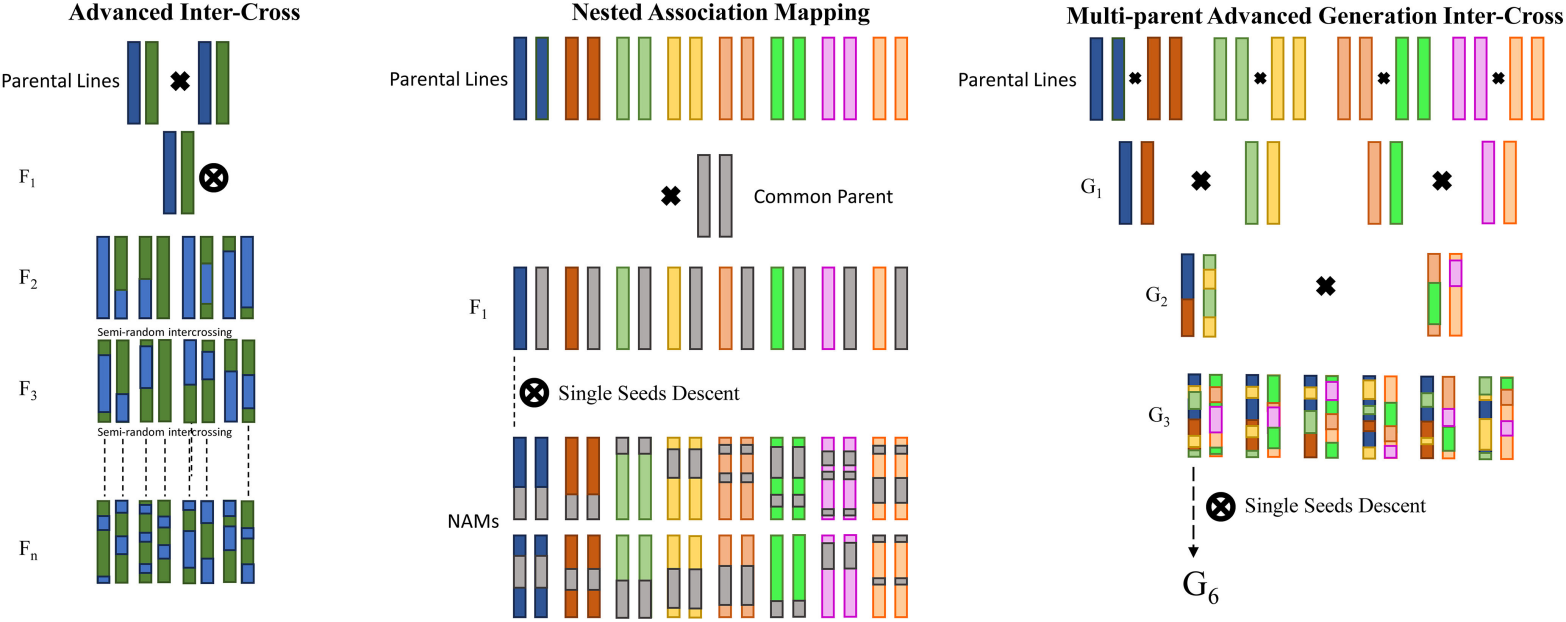
Graphical presentation of three advanced mapping approaches. From the left: advanced inter-cross (AIC), nested association mapping (NAM), and multi-parent advanced generation inter-cross (MAGIC).

Novel mapping strategies have emerged as alternatives to the conventional bi-parental populations for genetic mapping in crops, aiming to overcome the limitations associated with mapping precision and genetic diversity (Arrones et al., 2020; Scott et al., 2020; Yang et al., 2021). One such innovative approach is the advanced inter-cross (AIC; Rockman and Kruglyak, 2008) design, which entails the introduction of additional crossing generations before conducting genetic mapping. Despite its potential benefits, AIC has not been extensively adopted in crop studies, potentially due to the increased complexity it entails, and the labor-intensive manual crossing required for self-fertilizing species. Indeed, two main strategies have gained attention by the research community: nested association mapping (NAM; Yu et al., 2008) and multi-parent advanced generation inter-cross (MAGIC; Cavanagh et al., 2008) populations. As depicted in **Figure 1**, NAM populations involve crosses between a recurrent founder line and multiple alternative founders, akin to sets of bi-parental populations interlinked through a common parent, thereby making it conceptually familiar to researchers accustomed to working with bi-parental populations. While NAM populations offer increased genetic diversity, their approach to capturing greater genetic recombination largely relies on expanding the number of lines screened, much like bi-parental populations. In contrast, the MAGIC design represents a more intricate approach, building upon the principles of AIC. It involves the inter-crossing of several founders over multiple generations, followed by silting to generate inbred lines. Typically, MAGIC populations descend from a variable number of parents, commonly 4, 8, or 16, adhering to a straightforward funnel breeding design, and each line inherits alleles from all parents, resulting in the formation of random mosaics of founder haplotypes. By harnessing increased genetic recombination and genetic variation, MAGIC populations strive to overcome the limitations inherent to bi-parental populations in QTL mapping endeavors. The advantages offered by these novel mapping population strategies encompass heightened mapping power and resolution, expanded genetic diversity vis-à-vis bi-parental populations, and the minimal presence of population structure. Consequently, these strategies have witnessed an upsurge in popularity, leading to the development of multiple populations catering to various major crops. Their availability presents opportunities for QTL replication across diverse populations and enables the amalgamation of different populations to augment mapping power. However, it is worth noting that such populations are conceived with a long-term perspective, serving as enduring platforms for mapping non-target traits and exploring the genetic architecture of complex traits and the interrelationships between traits in the source population.

#### 6.3.1 Tomato

In 2015, Pascual et al. (2015) introduced the first tomato MAGIC population. Through resequencing of the founder genomes and their selection based on the obtained informative SNPs, they achieved a significant increase in recombination frequencies compared to biparental populations, facilitating haplotype prediction, and allowing QTL detection for fruit weight. Moreover, the combination of the MAGIC population with whole genome sequencing of the founders enabled the identification of candidate polymorphisms underlying QTLs, reducing the number of candidates from thousands to a handful. Using the same population, Diouf et al. (2018) assessed the impact of water deficit and salinity on fruit quality in contrasting environmental conditions. By evaluating 250 individual lines over two years, they identified QTLs for fruit quality and yield components, phenology traits, and a vegetative trait. The study mapped a total of 54 QTLs, with a considerable proportion (65%) being treatment specific. The MAGIC population facilitated high-resolution mapping and the projection of QTL intervals on the genome, enabling the identification of co-localized QTL regions. Candidate gene detection was performed based on the allelic effects of parental lines and sequence information, demonstrating the potential of the MAGIC population for dissecting the genetic basis of complex traits like fruit quality and elucidating genotype × environment interactions. Lastly, in a study by Bineau et al. (2021), the impact of high-temperature stress in tomato was investigated using both the MAGIC population developed by Pascual et al. (2015) and a core collection of small-fruited tomato accessions. The populations were evaluated for various yield components, phenology, and fruit quality traits under optimal and high-temperature conditions. The research demonstrated that high temperature significantly affected all traits, but a few genotypes with stable yield under high-temperature stress were identified. Plasticity indices were calculated to measure the extent of the heat impact on each trait. QTL analysis in the MAGIC and core collection populations led to the identification of a total of 69 plasticity QTLs associated with tomato heat response. Transcriptome analysis of the ovary in genotypes with contrasting heat responses revealed differentially expressed genes, providing insights into the molecular mechanisms underlying heat stress tolerance.

In 2019, Campanelli et al. (2019) further expanded the application of the MAGIC tomato population by leveraging it for the development of genotypes with desirable agronomic traits through Participatory Plant Breeding (PPB). Their MAGIC population was grown in an organic field experiment and, through phenotypic selection and molecular analysis, individuals with resistance genes and desired fruit shape were identified. Selected plants exhibiting desirable traits were used to generate stable lines in subsequent generations, providing an important genetic resource for the tomato scientific community. The MAGIC population was also cultivated in different organic farms, revealing significant phenotypic variations in development, productivity, and fruit color. This variability was harnessed to select tomato families adapted to specific environmental conditions, agricultural practices, and market requirements.

#### 6.3.2 Pepper

While only two eight-way pepper population obtained using landraces as parental lines has been reported as “in progress” state at the Universitat Politècnica de València (Arrones et al., 2020) and Sativa (www.sativa.it), in collaboration with University of Torino (Martina et al., 2023), without further reports, in tomato two MAGIC populations, originated by crossing eight founder genotypes, have been reported.

#### 6.3.3 Eggplant

Mangino et al. (2022) reported on the development of the first eggplant MAGIC population (S3MEGGIC), whose parental lines were previously genomically investigated by Gramazio et al. (2019). It consists of 420 individuals obtained by crossing seven cultivated eggplant varieties with one wild relative, *S. incanum*. After genotyping with eggplant SPET 5k probes GWA analysis revealed strong associations between specific genes involved in anthocyanin biosynthesis and the target traits, and two MYBs, similar to MYB113, a key transcription factor in the pathway, showed significant associations with anthocyanin presence in vegetative plant tissues (PA) and fruit epidermis (FA). Additionally, a COP1 gene encoding a photo-regulatory protein exhibited significant associations with the light-insensitive anthocyanin pigmentation under the calyx (PUC) trait. Comparing the eggplant genome with the tomato genome, duplication events of an ancestral MYB113 gene were detected and its translocation from chromosome 10 to chromosome 1 was identified. These findings provide insights into the evolutionary history of anthocyanin-related genes in eggplant. The S3MEGGIC population represents the largest recombinant population available for eggplant research. Its extensive genetic diversity and trait associations make it a powerful tool for future eggplant genetics and breeding studies.

#### 6.3.4 Potato

The polyploid nature of the potato genome, together with its tendency to inbreeding depression (Zhang et al., 2019), has reduced the possibilities of developing novel mapping approaches, and the only population that have been reported so far is a NAM population that uses M6, a diploid self-compatible inbred line *S. chachoense* harboring several resistances, including early blight (*Alternaria solani*), common scab (*Streptomyces scabies*), late blight (*Phytophthora infestans*), and cold-induced sweetening, as common progenitor (Jansky et al., 2014). However, recent developments in diploid self-compatible clones offer a promising solution to overcome inbreeding depression and replace the current out-breeding method with a true seed-based F_1_ hybrid system (de Vries et al., 2023). While this idea has been previously regarded as unrealistic, diploid breeding program has successfully produced self-compatible offspring by employing a homozygous progenitor with the *Sli* gene (Clot et al., 2020; Eggers et al., 2021). F_1_ hybrid potato breeding opens doors for the development of new potato products with desirable trait combinations, benefiting all stakeholders in the potato chain (Lindhout et al., 2011).

### 6.4 Phenomics and genomic prediction

Phenomics and genomic prediction (GP), followed by genomic selection (GS), are two groundbreaking approaches in the development of plant varieties (Zhao et al., 2019; Pasala and Pandey, 2020; Crossa et al., 2021, R2D2 Consortium et al., 2021; Budhlakoti et al., 2022; Parveen et al., 2023). Even if the vast amount of data provided by genotyping technologies has made phenotyping the current bottleneck in plant breeding, the deployment of remote sensing technologies has revolutionized phenotyping by enabling the collection of large volumes of precise and efficient data. By combining phenomic data with genomic prediction, which utilizes genome-wide marker datasets, researchers can significantly enhance the accuracy of yield predictions.

#### 6.4.1 Tomato

In 2020, Cappetta et al. (2020) reviewed the potentiality of genomic prediction in tomato, highlighting how implementing GP requires optimizing field trial management, agricultural practices, seed production, phenotyping, and sequencing, but also that a careful evaluation of parameters such as inbreeding levels, marker metrics, and the number of individuals to assess is essential. According to the authors, the integration of GP in the Single Seed Descendent scheme and backcrossing programs can reduce the number of generations and shorten the selection process in tomato, and the identification of candidate genotypes and undesirable segments through genotyping platforms can enhance trait introgression. Successively, Cappetta et al. (2021) applied GP on a F_4_ population for the prediction of yield and soluble solid content (SCC). The developed models exhibited a high prediction accuracy for both yield production (0.729) and SCC (0.715). Implementing these predictive models improved the efficiency of selection in subsequent breeding cycles, showcasing the potential of GS in expediting the breeding of heat-tolerant tomato varieties. Furthermore, by annotating SNPs located in gene body regions and conducting QTL analysis, the study identified five potential candidate genes involved in the response to high temperatures. This information was integrated into a GS model, utilizing a calibrated panel of markers.

By applying phenomic tools, Johansen et al. (2020) conducted field and UAV-based phenotyping of a wild tomato species to predict biomass and yield based on UAV imagery. Shape features derived from UAV, such as plant area and border length, were found to be important predictors. Multispectral UAV imagery collected 2 weeks prior to harvest produced the highest explained variances for biomass and yield. The study demonstrated the feasibility of predicting biomass and yield up to 8 weeks prior to harvest.

#### 6.4.2 Pepper

While no works have been previously reported in eggplant for these two novel applications, their potentialities were investigated in pepper by few researchers. Hong et al. (2020) evaluated the potential of genomic selection in predicting fruit traits such as length, shape, width, weight, and pericarp thickness. By using a core collection consisting of 302 C*. annuum* complex accessions and testing various genomic prediction models, the researchers identified the Reproducing Kernel Hilbert Space (RKHS) model as producing the highest prediction accuracies across the fruit traits. Such model was further validated using a separate population of recombinant inbred lines, achieving moderate prediction accuracies. Successively, Kim et al. (2022) focused on capsaicinoid contents in chili peppers and employed genomic selection to predict these traits. Capsaicinoids are responsible for the pungency of chili peppers, and their regulation has not been completely deciphered. The researchers utilized a core collection of 351 *Capsicum* accessions as a training population and 96 breeding lines as a testing population. They tested different numbers of genome-wide SNP markers and identified the optimal marker set for genomic selection, achieving the highest mean prediction accuracy. Through GWAS, they also identified markers associated with capsaicinoid biosynthesis genes and quantitative trait loci. Integrating these markers as fixed-effect markers improved the prediction models, leading to higher accuracies. This study laid the groundwork for developing pepper cultivars with different capsaicinoid levels using genomic selection for capsaicinoid contents.

On the phenomics side, Pereira-Dias et al. (2020) focused on the characterization of a diverse collection of *C. annuum* accessions from various regions in Spain, particularly the bell types known as Pimiento Morrón. By employing both conventional descriptors and high-throughput digital phenotyping using Tomato Analyzer software, the researchers evaluated 32 conventional traits and 35 digital traits. They found substantial variation in both types of traits, highlighting the diversity of Spanish peppers in terms of plant and fruit morphology. Digital phenotyping proved to be more accurate in discriminating varietal groups, with digital parameters outperforming conventional traits in distinguishing categories and revealing significant differences among closely related groups. Furthermore, a subset of four conventional descriptors and 13 Tomato Analyzer traits were identified as the most discriminant in distinguishing closely related *C. annuum* accessions. More recently, Fumia et al. (2023) investigated different methods of genomic and phenomic selection in the *Capsicum* core collection within the G2P-sol project, with the aim of identifying genotypes with heat tolerance traits for pepper breeding. By combining classical and multispectral phenotyping methods and integrating genomic data, they identified 33 promising genotypes for heat tolerance breeding, with 13 genotypes being selected by multiple methods. The study revealed that the combination of phenomic and genomic approaches yielded better selection results compared to individual methods. Interestingly, when each method was used independently, similar results were obtained, suggesting the robustness of the selection methods. This study exemplified the potential of utilizing high-throughput data, including phenomic and genomic information, to compensate for limited germplasm knowledge and overcome logistical or financial constraints in pepper breeding programs.

#### 6.4.3 Potato

The progression of genomic prediction techniques in tetraploid potato breeding is evident through a series of innovative studies. Initial research conducted by Wilson et al. (2021) signified a shift towards models that integrate dominance for certain traits, with an inclination towards non-parametric models to better capture complex genetic interactions. Selga et al. (2022) then scrutinized the predictive power of Bayesian Ridge Regression for genomic estimated breeding values, shedding light on the intricacies involved in early selection phases. Subsequent investigations by Ortiz et al. (2022) offered fresh insights into various genome-based prediction strategies, emphasizing the role of genotype-environment dynamics, and supporting both linear and kernel-based models for traits assessment. Advancing these concepts, Adams et al. (2023) assessed prediction models within diploid hybrid potato breeding, aiming to enhance genetic improvements across compound traits. The most recent contribution from Cuevas et al. (2022) involved examining both single-trait and multi-trait multi-environment models, underscoring the efficacy of multi-trait approaches in complex trait forecasting. This succession of research underscores a clear trend towards more nuanced and robust predictive methodologies that accommodate the inherent complexities within potato breeding programs.

Similarly, potato phenomics research has made significant strides in overcoming traditional limitations associated with labor-intensive and time-consuming phenotyping techniques. de Jesus Colwell et al. (2021) addressed these challenges by employing point cloud data obtained from low-cost UAV imaging to create 3D surface models of potato plant canopies. This approach facilitated detailed and accurate measurements of plant height distribution, canopy ground cover, and canopy volume throughout the growing season. The study identified distinct patterns of canopy development, including growth, lodging, maturity, and senescence, exemplified by varieties ‘Nadine’, ‘Bonnie’, and ‘Bounty’. This innovative method holds promise for alleviating current bottlenecks in plant development studies, enabling larger-scale population-based field studies in the future. Neilson et al. (2021) focused on the importance of potato tuber shape as a quality trait for breeding and variety development. They introduced an image analysis pipeline using commercially available cameras to extract tuber shape statistics, offering a more efficient alternative to manual measurements. The study successfully classified mean tuber shape from field-grown plants within ideal parameters for processing markets with high accuracy. The identification of parents with higher breeding value for tuber shape based on progeny performance demonstrates the potential for improving breeding selection processes. Johnson et al. (2021) addressed the urgent need for rapid and automated identification of blight disease in potatoes. They introduced an automated system utilizing Mask Region-based Convolutional Neural Network (Mask R-CNN) architecture for detecting blight disease patches on potato leaves in field conditions. The model, trained on a dataset of 1,423 images, demonstrated high precision in differentiating between diseased and healthy portions of the leaf, offering a practical solution for timely disease management in potato farming. Liu et al. (2021) highlighted the importance of accurate phenotyping for external quality attributes of potato tubers in breeding. They developed a 3D image analysis method for counting potato eyes and estimating eye depth, overcoming the limitations of subjective naked-eye visual evaluation. The study identified shape uniformity traits with better discriminatory power between varieties, demonstrating the potential of 3D image analysis in improving the accuracy of phenotyping complex geometrical traits in potato breeding.

## 7 Concluding remarks and future perspectives

**Figure 2:**
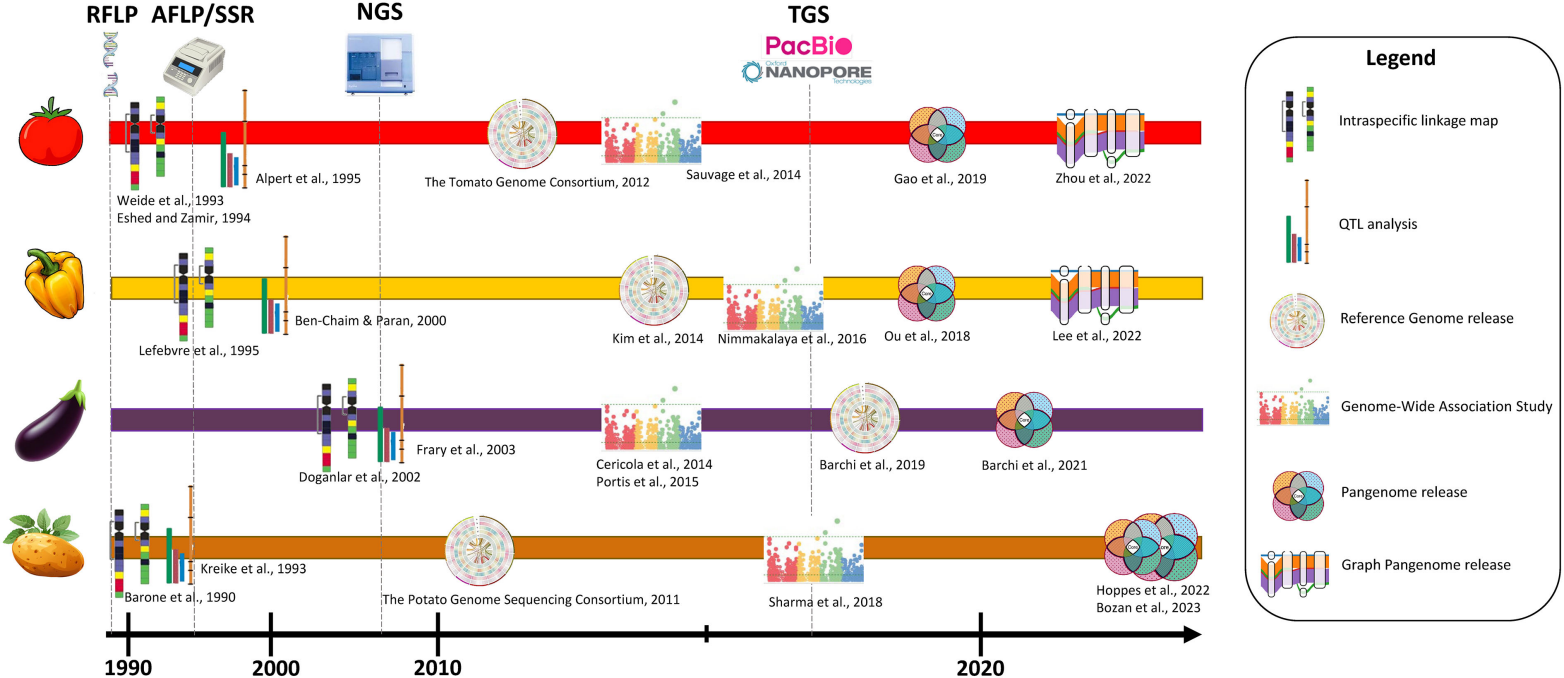
Overview of the evolution of the technologies and tools used in Solanaceae crops.

Altogether, research and breeding efforts have produced solid knowledge of Solanaceae genomics and genetics. Decades of varietal development have only tackled the surface of breeding for resilience traits, mainly focusing on diseases resistance and on the development of superior varieties, qualitatively fulfilling consumer liking and agronomically satisfying producers. Nowadays, it is pivotal to switch such tendencies, investing research efforts in the understanding of the resilience mechanisms to both biotic and abiotic stressors already available in the genebank-preserved germplasm. By reviewing the state of the art of the -omics research on Solanaceae, as summarized in **Figure 2**, with a special focus on research for resilient traits, our work provides a starting point for the development of the future experimental designs.

## Author contributions

MM: Writing – review & editing, Writing – original draft. VDR: Writing – original draft, Writing – review & editing. GM: Writing – original draft, Writing – review & editing. AA: Writing – review & editing. LB: Writing – review & editing. GB: Writing – review & editing, Conceptualization. EDP: Writing – review & editing, Conceptualization. AV: Conceptualization, Writing – review & editing, Project administration. EP: Conceptualization, Writing – review & editing, Supervision.
